# Exploring Chronic Hypocalcemia: Insights into Autoimmune Polyglandular Syndrome Type 1—A Case Study and Literature Review

**DOI:** 10.3390/jcm13082368

**Published:** 2024-04-18

**Authors:** Giorgiana-Flavia Brad, Delia-Maria Nicoară, Alexandra-Cristina Scutca, Meda-Ada Bugi, Raluca Asproniu, Laura-Gratiela Olariu, Iulius Jugănaru, Lucian-Ioan Cristun, Otilia Mărginean

**Affiliations:** 1 Department XI Pediatrics, Discipline I Pediatrics, ‘Victor Babes’ University of Medicine and Pharmacy of Timisoara, 300041 Timisoara, Romania; brad.giorgiana@umft.ro (G.-F.B.); scutca.alexandra@umft.ro (A.-C.S.); asproniu.raluca@umft.ro (R.A.); olariu.laura@umft.ro (L.-G.O.); juganaru.iulius@umft.ro (I.J.); marginean.otilia@umft.ro (O.M.); 2 1st Department of Pediatrics, Children’s Emergency Hospital ‘Louis Turcanu’, 300011 Timisoara, Romania; bugi.ada@umft.ro; 3 Research Center for Disturbances of Growth and Development in Children BELIVE, ‘Victor Babes’ University of Medicine and Pharmacy of Timisoara, 300041 Timisoara, Romania; 4 Ph.D. School Department, ‘Victor Babes’ University of Medicine and Pharmacy of Timisoara, 300041 Timisoara, Romania; lucian.cristun@umft.ro

**Keywords:** chronic hypocalcemia, *AIRE* (autoimmune regulator) gene mutations, autoimmune polyendocrine syndrome type 1, autoimmune endocrinopathies, autoimmune diseases

## Abstract

Hypocalcemia is a common occurrence in pediatric patients, attributed to various causes and presenting with diverse clinical manifestations. A prompt evaluation is necessary to determine its underlying cause, whether it presents acutely or chronically, and to tailor treatment based on its severity. Among the potential causes of chronic hypocalcemia, primary hypoparathyroidism stands out. The case of a seven-year-old male patient with hypocalcemia reported in this article serves as an illustration, wherein targeted next-generation sequencing revealed a homozygous p.R257X mutation in the *AIRE* gene, indicative of autoimmune polyendocrine syndrome type 1 (APS-1). It poses challenges due to its multisystemic nature and involvement of specific autoantibodies, often leading to underdiagnosis, owing to its rarity, varied manifestations, and incomplete penetrance. A comprehensive review of the APS-1 literature was conducted to provide insights into the clinical manifestations, genetic spectrum, potential immunological mechanisms, and current medical strategies. Additionally, the recognition of *AIRE* gene mutations is crucial for facilitating genetic diagnosis, prognosis, and potential treatment strategies for APS-1. The management of such cases involves individualized approaches to treatment, regular monitoring, medication adjustments, and the early identification of associated conditions.

## 1. Introduction

Calcium is the most abundant mineral in the human body and plays a vital role in numerous biological functions, such as blood coagulation, membrane stability, muscle function, nerve activity, and bone mineralization. The normal serum calcium level is typically regulated by three key hormones including parathyroid hormone, vitamin D, and calcitonin. These hormones exert specific effects on the bowel, kidneys, and bones to ensure calcium homeostasis, with the support of the calcium-sensing receptor.

Hypocalcemia is a common clinical scenario in pediatric practice with multiple causes and clinical manifestations that may involve almost any organ system [1].

Several factors impact the clinical presentation of hypocalcemia, including the severity and timing of serum calcium reduction (acute versus chronic), the patient’s age, complications, and comorbidities.

Hypocalcemia may present as a medical emergency or a chronic diagnostic dilemma, with manifestations varying in severity from asymptomatic to potentially acute life-threatening crises, whose diagnosis and management require meticulous evaluation by clinicians.

A decreased serum calcium level is often encountered in an acute critical state with a multifactorial etiology, including abnormal PTH secretion, hypomagnesemia, sepsis, specific medications, and low dietary calcium intake. It manifests as a temporary condition, resolving upon the treatment of the underlying cause, or it may be incidentally discovered in asymptomatic children. The condition can be revealed by the assessment of Chvostek’s and Trousseau’s signs [2], indicators of both forms of hypocalcemia, or presented with symptoms such as muscle cramps, tetany, and laryngospasm. Additionally, arrhythmias such as torsade de pointes, ventricular tachycardia, and ventricular fibrillation, secondary to the prolongation of the QT interval [1], may indicate its presence.

Occasionally, hypocalcemia can become chronic or even lifelong as a consequence of irreversible damage to the parathyroid glands after surgery, or it indicates an early presentation of a genetic syndrome or is secondary to autoimmune destruction. It is usually the consequence of insufficient levels or resistance to PTH or vitamin D or a defect of the calcium-sensing receptor [3]. Renal calcification or injury, brain calcifications, cataracts, or paresthesia are suggestive signs of chronic hypocalcemia [4,5].

Our article aims to showcase a pediatric patient with chronic hypocalcemia diagnosed with autoimmune polyendocrine syndrome type 1 (APS-1), confirmed genetically, serving as the focal point for a narrative review. This review will emphasize particular aspects of APS-1, including its clinical manifestations, genetic spectrum, and potential immunological pathogenesis. Our goal is to improve the early recognition of APS-1 and the understanding of current therapeutic options.

The patient’s parents gave informed consent to publish anonymized details in this case report, and the ethical committee of the hospital approved the publication of this case.

## 2. Case Report

### 2.1. Patient’s Medical History

**Figure 1:**
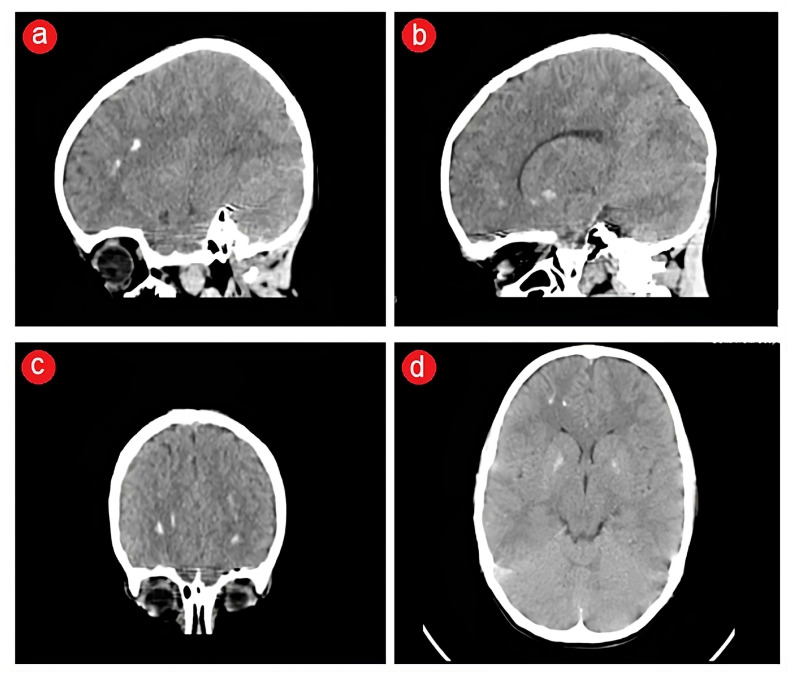
CT scan displaying calcifications in the frontal cortex and basal ganglia, with no indications of infarction, hemorrhage, or mass effect, as observed in sagittal (**a**,**b**), coronal (**c**), and axial (**d**) views.

A seven-year-old male patient was admitted to the Endocrine Department of tertiary care, Children’s Emergency Hospital in Timisoara, Romania, due to hypocalcemia detected during routine blood investigations. Recently, he was diagnosed with symptomatic focal epilepsy manifested as recurrent polymorphic motor seizures in the absence of the fever, with a typical electroencephalogram aspect. The patient underwent a cerebral computed tomography scan to investigate the causes of these recurrent seizures, which revealed diffuse basal ganglia and frontal cortex calcifications without evidence of infarction, hemorrhage, or mass effect (Figure 1). Despite receiving optimal doses of anticonvulsant drugs (valproic acid and levetiracetam), the patient remained unresponsive to treatment, presenting several daily seizure episodes lasting no longer than two minutes, often recovering spontaneously without any therapeutic intervention.

The patient was the first child of the couple, born from non-consanguineous parents, with an uncomplicated birth history and normal development. There was no significant family medical history of seizures, thyroid disease, or autoimmune disorders, and neither parent had any comorbidities. His younger sister was in good health and had no history of disorders.

### 2.2. Symptoms and Clinical Findings

**Figure 2:**
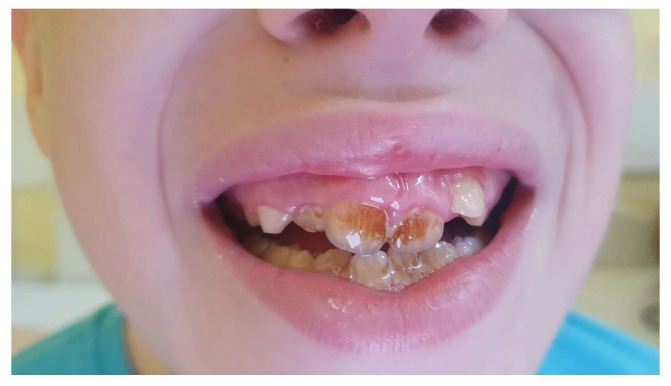
Patient’s dentition with enamel dysplasia.

Upon hospital admission, the patient presented dry, harsh skin, thin hair, and brittle and fragile nails, with vertical ridges suggestive of early onychodystrophy. Additionally, he had teeth enamel dysplasia (Figure 2). The patient frequently experienced muscle cramps in the foot and calves. A clinical examination pointed out positive Chvostek’s and Trousseau’s signs. Although no dysmorphic features were described, the patient had a history of delayed growth (BMI < 5th percentile for age and sex; SDS for height = −1.8).

### 2.3. Biological and Paraclinical Assessment

The constellation of clinical findings prompted further biological investigations, revealing a decreased serum ionized calcium level of 0.73 mmol/L (1.05–1.3 mmol/L), a serum total calcium level of 1.69 mmol/L (2.3–2.75 mmol/L), intact PTH < 4.6 pg/mL (18.5–88 pg/mL), and an increased serum phosphorus level of 3.72 mmol/L (1.1–2 mmol/L). A decreased urine calcium level of 0.38 mmol/24 h (1.75–7.5 mmol/24 h) and a urine phosphorus level of 7.73 mmol/hours (13–42 mmol/24 h) were encountered too. The serum concentrations of 25-hydroxyvitamin D, 1,25-dihydroxyvitamin D, magnesium, alkaline phosphatase, albumin, and protein were within normal limits. However, an evaluation of anti-parathyroid and anti-calcium-sensing receptor antibodies was not possible as these investigations were not available in Romania at that time.

**Figure 3:**
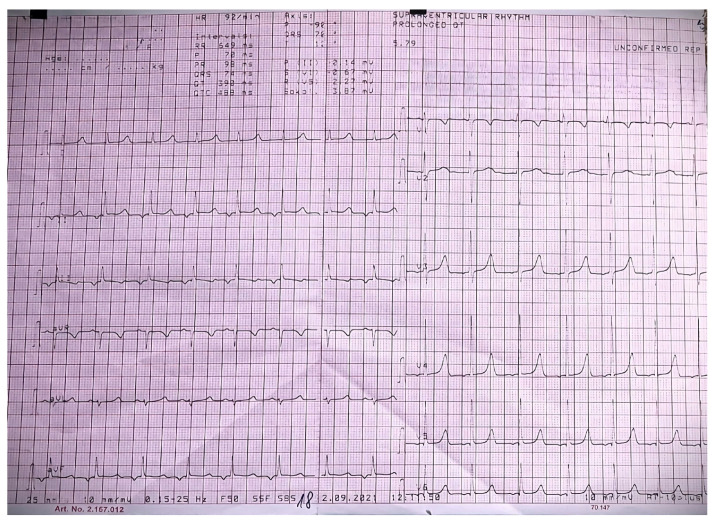
Patient’s EKG aspect: prolonged QT interval and peaked T waves secondary to hypocalcemia.

**Figure 4:**
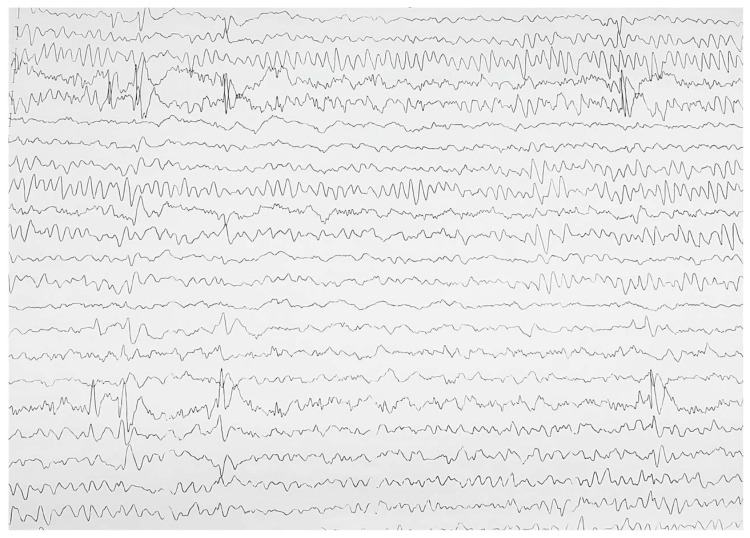
Patient’s EEG aspect.

The patient underwent various evaluations. The prolongation of the QT interval (Qtc = 488 msec and normal <450 msec) and peaked T waves were shown on the electrocardiogram secondary to hypocalcemia (Figure 3). At the same time, the needle electromyography revealed spontaneous activity in the form of doublets and triplets at a rate of 4–15 Hz, suggestive of acute tetany. Bilateral symmetrical circular corneal opacities were described at the ophthalmologic examination. A more recent neurological reevaluation occurred, and the patient’s EEG displayed similarities to the previous examination, leading to recommendations for continuing the previously initiated anticonvulsant medication (Figure 4).

A preliminary diagnosis of primary hypoparathyroidism was considered based on the clinical manifestations (onychodystrophy and teeth enamel dysplasia, muscle cramps, brain diffuse calcifications, and bilateral cataracts), typical aspects of the electrocardiogram and electromyography, as well as the results of the blood investigations. No particular complications associated with primary hypoparathyroidism were encountered.

To further explore the genetic basis, screening was performed using the Illumina TruSight One Sequencing Panel, providing a comprehensive coverage of clinically relevant genes. The targeted next-generation sequencing revealed a stop-gain variant mutation p.R257X (c.769C > T) in homozygous status in the *AIRE* gene on chromosome 21q22.3, highly suggestive of autoimmune polyendocrine syndrome type 1 (APS-1). Subsequent family genetic screening indicated that each member was heterozygous for the same mutation.

Various biological investigations were performed to explore other possible autoimmune endocrinopathies or diseases associated with APS-1 in this patient.

The thyroid gland was evaluated by testing the thyroid hormones and specific thyroid antibodies (anti-thyroid peroxidase and anti-thyroglobulin antibodies), which were within normal limits. Addison’s disease was ruled out (normal baseline cortisol and baseline ACTH) and negative steroid 17- and 21-hydroxylase antibodies. Additionally, a possible immunodeficiency was excluded by normal values obtained from T-lymphocyte proliferation studies (total lymphocytes = 2885/µL (1500–6500/µL) and CD4+ Th lymphocytes = 1406/µL (400–2500/µL).

The additional blood investigations performed provided results within normal values for the patient’s age (Table 1). These findings ruled out the association of several autoimmune endocrinopathies or autoimmune diseases, including celiac disease, autoimmune hepatitis, inflammatory bowel disease, irritable bowel disease, pernicious anemia, type I diabetes mellitus, or B12 deficiency.

The examination of the gastrointestinal system revealed no abnormalities or the presence of *Candida albicans* infection, with normal mucous aspects described at upper endoscopy and colonoscopy.

### 2.4. Therapeutic Intervention

To correct the severe hypocalcemia and to prevent the recurrence of new episodes of hypocalcemic seizures, the patient received high doses of intravenous calcium gluconate infusion (4–5 g per day) for three weeks to stabilize calcium levels.

As serum calcium concentration improved and remained stable, the patient began receiving an oral supplementation of calcium carbonate, along with vitamin C and vitamin D to enhance intestinal calcium absorption. Medication doses were carefully adjusted over time based on the biological laboratory findings to nearly normalize the serum calcium level (6–7 g elemental calcium, 1 μg oral calcitriol, and 2000 IU cholecalciferol daily), alongside the administration of anticonvulsant medications (valproic acid and levetiracetam). The patient was advised to use oral nystatin solution for oral candidiasis to prevent systemic *Candida albicans* infections. Additionally, a strict low-phosphorus diet was recommended. This treatment regimen was well tolerated by the patient, with no episodes of recurrent seizures reported.

### 2.5. Follow-Up and Outcome

The patient underwent regular check-ups every 3–4 months, involving detailed clinical examinations, standard blood tests, and necessary adjustments to the prescribed medications. Fortunately, no additional conditions were identified during these visits. However, eight months after the APS-1 diagnosis, the patient developed severe oral candidiasis following a bacterial infection that was treated with oral antibiotics. Treatment included intravenous infusion and the topical application of fluconazole, which resulted in a favorable response.

During this episode, the mother recalled a vague history of multiple episodes of oral candidiasis since infancy, often occurring after short-term antibiotic therapy. Remarkably, the family had considered this recurrent condition as normal. This revelation underscored the existence of chronic oral mycosis in the patient’s life, which is strongly associated with APS-1. As a result, antifungal prophylaxis was initiated to prevent *Candida albicans* infections in the future.

## 3. Discussion

### 3.1. Literature Review

#### 3.1.1. Definition of APS-1

Autoimmune polyglandular syndrome (APS) represents a designated group of sequential or simultaneous disorders characterized by immune dysregulation and the coexistence of at least two endocrine gland deficiencies mediated by autoimmune mechanisms and, in some cases, by immunodeficiency [6]. Many non-endocrine organs, such as the respiratory and digestive tract, gonads, hair, or skin, might be affected, too, by developing non-endocrine autoimmune diseases.

The first classification of APS was proposed by Neufeld and Blizzard in the 1980s and consisted of four main forms [7]. However, it was changed later and categorized into three major syndromes: autoimmune polyglandular syndrome type 1 (APS-1), autoimmune polyglandular syndrome type 2 (APS-2), and immune dysregulation, polyendocrinopathy, enteropathy, X-linked (IPEX) syndrome [8]. Although they are phenotypically different, these autoimmune disorders share similar immune and genetic defects, such as the concomitance of polyautoimmunity or multiple autoimmune syndromes and familial autoimmunity. This situation is called “autoimmune tautology” [9].

According to the 2022 update of the International Union of Immunological Societies, APS-1 and IPEX were considered to be diseases of immune dysregulation [10]. These syndromes are monogenic disorders caused by mutations in the autoimmune regulator (*AIRE*) and forkhead box protein P3 (FOXP3) genes, respectively, while APS-2 is a polygenic, complex genetic disorder with a solid connection to the human leukocyte antigen locus and with an intricate inheritance pattern [11].

Out of the existing major forms, APS-1, also known as autoimmune polyendocrinopathy–candidiasis–ectodermal dystrophy, is a rare autosomal recessive condition consisting of the classic triad of disorders including chronic mucocutaneous candidiasis, hypoparathyroidism, and autoimmune adrenal insufficiency (Addison’s disease).

While APS-1 is characterized by the presence of the triad, it also can engage additional endocrinopathies caused by autoimmune tissue disruptions as well as ectodermal dysplasia and non-endocrine immune diseases [11,12].

According to the revised definition, APS-1 diagnosis requires the existence of at least two diseases of the characteristic triad. If there is a family history of this syndrome, only one triad component is necessary for the diagnosis. Essential diagnostic tools for APS-1 comprise genetic studies to identify the carriers of pathogenic *AIRE* mutations and anti-interferon antibodies [13].

#### 3.1.2. Epidemiology

Worldwide epidemiological studies of APS-1 depicted a decreased overall prevalence, with a mean of 10 cases per one million population [14]. The highest prevalences were reported in genetically restricted ethnic groups or populations with a higher degree of consanguinity. For instance, the Iranian Jewish population had a prevalence of 1 case per 9000 individuals [15], Finns had 1 case per 25,000 individuals [16], and Slovenians [17] had 1 case per 43,000 individuals, while in contrast, APS-1 had the lowest prevalence in France (1 case per 500,000 population) [18] and Japan (1 case per 10 million population) [19]. In the United States, its prevalence varied between 1 case per 100,000 and 300,000 individuals [12].

Studies pointed out a significant discrepancy in the onset of clinical manifestations between European and American patients with APS-1. In European patients with APS-1, 80–90% presented with one or more triad manifestations at onset [20], while 40–80% of American patients had non-major diseases at the onset (urticarial eruption, autoimmune hepatitis, autoimmune gastritis, autoimmune pneumonitis, and Sjögren-like syndrome), with a delayed development of classical manifestations [21].

#### 3.1.3. Pathogenesis and Autoimmunity of APS-1

The discovery of the involvement of mutations of the *AIRE* gene in the APS-1 was described in 1997 [22], followed by the elucidation of its immunological functions within the thymus in 2002 [23]. The gene is situated on chromosome 21q22.3 [24].

*AIRE* is a multidomain protein comprising three domains: a nuclear localizing signal domain at the N-terminus, the SAND domain in the middle responsible for DNA binding, and at the C-terminal, two zinc fingers, PHD1 and PHD2, crucial for binding DNA and interacting with other proteins [25].

It is a transcription factor primarily expressed in the thymic medullary epithelial cells, playing an important role in self-antigen presentations. Reduced levels also can be encountered in secondary lymphoid organs such as lymph nodes, tonsils, spleen, fetal liver, and peripheral blood cells. Additionally, *AIRE* serves as a proapoptotic factor, influencing the final maturation stage of these thymic medullary cells [26].

Mutations in the *AIRE* gene can induce the production of anti-interferon-ω and -α antibodies. Interestingly, despite the presence of these antibodies, patients with APS-1 do not show an increased susceptibility to viral infections [27]. It is considered that these antibodies appear years before the onset of the first clinical manifestation and are valuable diagnostic screening markers for the detection of APS-1 [27].

*AIRE* encodes a DNA binding protein known as the “autoimmune regulator” or “thymus-enriched transcription factor” highly expressed in the thymus gland, generating naive autoreactive T-lymphocytes. This protein is engaged in central immune tolerance and prevents autoimmunity by controlling the expression of several thymic proteins, regulating the presentation of autoantigens, and facilitating the negative selection of autoreactive T-lymphocytes in the thymus [28]. Further, it is involved in the control of thymic T-lymphocyte tolerance to peripheral antigens, contributing to the induction of T-lymphocyte tolerance and influencing the maturation and function of peripheral dendritic cells [24].

The *AIRE*-mediated expression of peripheral tissue antigens drives the thymic development of a naturally occurring subset of organ-specific T(regs). These are identified as CD4+Foxp3+, playing critical roles in the prevention of autoimmunity, the maintenance of immune homeostasis, and the suppression of anti-tumor immune responses [29,30]. Their functional role in cancer progression was suggested in human cancers by the density of T(regs) within tumor lesions, predicting poor clinical outcomes [31].

The decreased expression of the “autoimmune regulator protein” resulting from *AIRE* mutations, as described in APS-1, induces a reduced presentation of self-antigens by medullary thymic epithelial cells and dendritic cells for the development of T-lymphocytes. This alteration can affect the endocrine glands and may cause tissue-specific autoimmunity due to the defective elimination of autoreactive T-lymphocytes [14]. Ultimately, it triggers the autoimmune destruction of target organs by disrupting immunological tolerance [32].

The thymic escape of autoreactive T-lymphocytes was described in patients with APS-1. They developed various autoantibodies against different autoantigens including self-antibodies against Th17-associated cytokines, antibodies targeting glutamic acid decarboxylase 65, or those against NALP5 [33]. Furthermore, T-lymphocytes and Th17-associated cytokines (defective IL-17A, IL-17F, and IL-22 responses to *Candida albicans* antigens) responsible for the defense against fungal infection emerged from an early age [34]. Remarkably, despite these immune dysregulations, the B-lymphocyte response to *Candida albicans* remained normal, preventing the development of systemic candidiasis.

Therefore, *AIRE* serves a dual role in immune tolerance maintenance by enabling the deletion of autoreactive T-lymphocytes and fostering the development of Foxp3+ T(regs).

#### 3.1.4. Correlation between Genotype and Phenotype in APS-1

Recently, Oftedal et al. [35] proposed a revised classification of APS-1 based on the various spectrum of phenotypes related to *AIRE* mutations and included two major forms:

(1) “classical APS-1” is characterized by recessive inheritance (homozygous or compound heterozygous mutations of the *AIRE* gene), the classical loss of function mechanism being the typical mechanism [36]; it consists of at least two of the three main disorders of the triad, complete penetrance, and positive interferon antibodies.

(2) “non-classical APS-1” consists of dominant heterozygous *AIRE* mutations located in the PHD1, PHD2 zinc finger, and in the SAND, acting with a dominant negative effect, reducing the effectiveness of the wild-type allele and transactivation of downstream genes. The mono-allelic *AIRE* p.G228W or pR247C mutations have been identified to act in a dominant fashion and could provide insights into the structure–function relationship of the *AIRE* protein [35,36,37]; these mutations have a milder autoimmune phenotype, with fewer autoimmune manifestations or unaffected patients by autoimmunity. This suggests an incomplete clinical penetrance and with late-onset and less positive interferon antibodies. Familial clustering with a later onset points out a possible modulation of the phenotypic expression of common organ-specific autoimmune diseases [38,39].

To date, approximately 186 single-nucleotide mutations or large deletions have been identified within the exon/intronic sequence of the *AIRE* gene [40]. Specific mutations in this gene are prevalent within certain populations and are often associated with particular geographic regions. Notable mutations include those located in exons 2, 6, 8, and 10, resulting in the transcription of a truncated protein [41].

The most common *AIRE* gene mutation is a nonsense mutation p.R257X (c.769C > T) on exon 6, prevalent in the Finnish population and detected in 87% of patients (“Finnish major mutation”) [16]. This mutation is also found at a high frequency in Turkish, Italian, Russian, Ukrainian, and Serbian populations, accounting for 75% of alleles in Central and Eastern European APS-1 patients [35,42].

Other frequent *AIRE* mutations include the Y85C missense mutation (c.254A > G) in the Iranian Jewish population, deletion 967-979del13 in Norwegians, British people, and North Americans, and C322fsX372 (del13 in exon 8) mutations in Anglo-Saxon populations [43,44,45,46,47]. Heterozygous *AIRE* mutations, notably p.L323SfsX51 followed by p.R257X, were frequently observed in American APS-1 patients [22].

The relationship between the genotype and phenotype is complex and varies among populations. For example, the p.R257X mutation in Finnish patients was correlated with chronic mucocutaneous candidiasis, while Addison’s disease and chronic mucocutaneous candidiasis had a low incidence in the Iranian Jewish population with the Y85C missense mutation [42]. In contrast, the autosomal dominant G228W missense mutation is typically found in patients with autoimmune thyroiditis [36]. Alopecia areata is more common in individuals with deletion 967-979del13 [48], and certain mutations like p.L323SfsX51 have been associated with the development of certain non-endocrine autoimmune manifestations such as pneumonitis and hepatitis [49,50]. Recently, two protein-coding *AIRE* mutations, specifically rs74203920 and rs2075876, were linked to autoimmune Addison’s disease [36,51].

Despite APS-1 being a monogenic autoimmune disease, there is significant variability in clinical manifestations with a weak correlation between genotypes and clinical phenotypes. This may be the result of complex interactions between various factors, including genetic, epigenetic, immunological, molecular, and/or environmental factors (socioeconomic factors, lifestyle, and habits). Additionally, there may be an unknown third factor that could influence both the clinical manifestation and the progression of this disease [12,21].

Regarding the patient described in this article, although the anti-interferon antibodies could not be tested, the presence of the homozygous mutation in the *AIRE* gene (p.R257X) confirmed the diagnosis of APS-1 according to the new criteria.

The genetic evaluation revealed the existence of a homozygous mutation (p.R257X) in this patient, consistent with characteristics seen in the Finnish population [16]. Furthermore, the p.R257X mutation of the *AIRE* gene is the most commonly encountered in the population of Eastern and Central Europe, which illustrates the prevalence of this mutation in our region. The identification of this mutation was correlated with chronic mucocutaneous candidiasis, proving the correlation between genotypes and phenotypes in this patient.

As for limitations, this patient represents the only published case of APS-1 in Romania, making it challenging to draw comparisons with similar cases. Although there are diagnosed cases, the absence of published comparable patients complicates the assessment of clinical manifestations at onset, therapeutic management, the exploration of the spectrum of *AIRE* mutations, and the establishment of genotype–phenotype correlations within the Romanian population context.

#### 3.1.5. Clinical Features of APS-1

The onset age of the initial manifestation varies among the triad components. Chronic mucocutaneous candidiasis usually starts presenting early during infancy, followed by hypoparathyroidism, which tends to appear previously in girls. The symptoms of Addison’s disease are the last to manifest and typically appear later, 5–10 years after hypoparathyroidism appears [52].

Chronic mucocutaneous candidiasis, the hallmark of APS-1, is the manifestation that usually appears and recurs in the first years of life in 70% of cases [53]. It may involve the skin, nails, and oral, anal, and genital mucosa and is secondary to the selective immunological deficiency of a normal T-lymphocyte-mediated response. Recurrent candidiasis should be investigated for the presence of coexisting endocrine disorders as other components of the APS-1 tend to develop later.

Hypoparathyroidism often emerges as the first endocrine gland dysfunction in patients with APS-1, presenting early with symptoms such as muscle cramping and spasms, paresthesia, neuro-muscular hyperexcitability tetany, and, in severe cases, seizures.

The dysfunction of the parathyroid glands leads to hypocalcemia and hyperphosphatemia, with low parathyroid hormone levels in the presence of antibodies against parathyroid autoantigens, such as the calcium-sensing receptor, NACHT leucine-rich repeat protein 5, NALP5, or organ-specific antibodies [54].

Adrenocortical insufficiency, commonly known as Addison’s disease, is the second endocrine disorder to occur in APS. It usually appears after the age of six, with an incidence peak around the age of twelve years. It is characterized by the insufficient production of glucocorticoid and mineralocorticoid associated with positive autoantibodies against antigen-targeting adrenal enzymes, including side-chain cleavage enzymes, 17-alpha-hydroxylase, and 21-hydroxylase. Notably, these antibodies can be detected in up to 48% of children presenting with components of APS-1, even before overt adrenal insufficiency, indicating an increased risk of developing this pathology [55].

The term ectodermal dystrophy refers to the particular aspect of the nails (onychodystrophy), dental enamel (teeth enamel hypoplasia), hair (alopecia), corneas (keratopathy), and skin (vitiligo) that may be described in some patients with APS-1. The responsible pathological mechanisms are variable and consist of specific immune mechanisms in the case of alopecia and vitiligo or chronic candidiasis for nail deformities.

Patients diagnosed with APS-1 may develop various autoimmune disorders caused by autoimmune tissue disruption, including endocrinopathies such as autoimmune thyroid disease, type 1 diabetes mellitus, primary hypogonadism, hypophysitis, or gastrointestinal manifestations comprising celiac disease, autoimmune hepatitis, early-onset pernicious anemia resulting from atrophic gastritis, and chronic diarrhea leading to malabsorption [11,12]. As these patients frequently present with a combination of multiple endocrine disorders, it is necessary to highlight the significance of screening for the associated conditions.

Pediatricians should be aware of the less common non-endocrine immune diseases of APS-1 in children for proper medical interventions. These may include conditions such as cancer (especially squamous cell carcinoma) asplenia, cholelithiasis, chronic inflammatory demyelinating polyneuropathy, chronic lung disease, and gastrointestinal dysfunction [56,57].

Typically, the detection of hypocalcemia in routine tests prompts an extensive series of investigations aimed at accurately evaluating calcium homeostasis. This becomes particularly vital in pediatric cases when the patient exhibits severe symptoms like afebrile recurrent seizures, arrhythmia, kidney stones, or brain calcifications detected on imaging. In such scenarios, a series of evaluations are conducted to uncover the underlying factors. Similar circumstances arose in the presented patient, where these evaluations performed pointed to chronic hypocalcemia due to primary hypoparathyroidism.

At the initial hospital admission, our patient displayed symptoms typical for ectodermal dystrophy and autoimmune hypoparathyroidism, significant components of APS-1. These manifestations are similar to those described in European populations, where APS-1 often manifests with major diseases at the onset, contrary to American patients who tend to present with non-major diseases initially. Additionally, the patient presented with onychodystrophy and teeth enamel dysplasia suggestive of ectodermal dystrophy, while the circulating target organ antibodies (including anti-thyroid peroxidase antibodies, anti-thyroglobulin antibodies, and steroid 17- and 21-hydroxylase antibodies) were negative.

A study by Skrabic et al. conducted in Croatia reported that onychodystrophy was identified as the first manifestation of APS-1 in most of the analyzed patients [58] in contrast with other studies [35,59]. This led the authors to propose onychodystrophy as a warning sign of APS-1 and a potential trigger for early screening. In the case presented, the patient’s mother could not accurately recall which was the first symptom that appeared.

#### 3.1.6. Therapeutic Approach in APS-1

The management of endocrinopathies of APS-1 requires appropriate hormonal replacement treatment. For patients with Addison’s disease, long-term glucocorticoid and mineralocorticoid replacement therapy is necessary, using daily hydrocortisone and fludrocortisone. Additionally, thyroid substitution with levothyroxine is indicated when hypothyroidism is associated, but it is initiated only after the initiation of glucocorticoid substitution therapy to prevent the precipitation of an adrenal crisis [60,61].

The current therapeutic approaches for autoimmune hypoparathyroidism typically involve administering calcium intravenously during acute tetany episodes, followed by oral calcium supplementation to ease clinical symptoms, manage hypocalcemia, and prevent the development of hyperphosphatemia and hypercalciuria. Additionally, active vitamin D analogs such as calcitriol are recommended as hyperphosphatemia and parathyroid hormone deficiency can affect the activation of 25-hydroxyvitamin D by 1-alpha-hydroxylase. However, conventional treatment may lead to potential short-term and long-term complications, including hypercalcemia, hypercalciuria, nephrocalcinosis, impaired renal function, and ectopic soft tissue calcification [62].

The physiological therapy involves hormonal substitution with recombinant human PTH (rhPTH), although it is not commonly used. Two molecule types are available for human use, the full-length rhPTH^1–84^ (less studied) and the intensively researched rhPTH^1–34^ analog, which contains the first 34 amino-terminal amino acids of the natural PTH molecule and is approved by the FDA and EMEA for poorly controlled hypoparathyroidism as a long-term therapy. Unfortunately, there are no comparative studies between the two rhPTH molecules to ascertain which one is more suitable for pediatric prescriptions [63].

Several studies reported their experience with rhPTH^1–34^ use in children and highlighted its effectiveness and safety in pediatric cases of hypoparathyroidism. This medication promptly corrects metabolic abnormalities associated with hypoparathyroidism, with a reduction in calcium supplements and activated vitamin D analog doses, thereby decreasing the frequency of tetanic episodes. Notably, the use of rhPTH^1–34^ demonstrated no treatment-related side effects or significant complications, improving the patient’s quality of life [64,65].

Winer et al. recommended the utilization of rhPTH^1–34^ as a twice-daily or thrice-daily subcutaneous injection regimen for improved metabolic control. However, pump therapy offers a more physiologically stable calcium homeostasis with minimal fluctuations, especially beneficial for children with congenital hypoparathyroidism and patients facing treatment failures like hypocalcemic seizures. Pump treatment induces a more natural rhythm of parathyroid hormone, leading to superior metabolic control with lower substitution doses [66,67]. It is a safe and effective replacement therapy as it reduces the variation in serum calcium levels even a lower total daily rhPTH^1–34^ dose.

The use of rhPTH poses a theoretical risk of osteosarcoma development as suggested by an initial animal study in which half of the Fisher rats receiving an increased dose of medication developed this type of bone cancer [68]. Consequently, the FDA prohibits the use of rhPTH in patients with open epiphyses.

Despite the initial concerns, both the Osteosarcoma Surveillance Study and the Forteo Patient Registry have not found any association between rhPTH treatment and the development of osteosarcoma among their patients [69,70]. Additionally, long-term studies on pediatric rhPTH treatment have not reported any occurrences of osteosarcoma [69].

Chronic mucocutaneous candidiasis consists of a chronic or recurrent infection caused especially by *Candida albicans*, affecting multiple areas along the gastrointestinal tract. To prevent this condition, medical experts recommend maintaining meticulous oral hygiene to reduce the risk of *Candida albicans* infection. Long-term strategies include using both toothpaste and chlorhexidine solution at bedtime. To effectively manage the infection, topical polyene drugs like nystatin suspension and amphotericin B are typically prescribed for 4–6 weeks or at least one week after the symptoms have resolved. To prevent the emergence of Candida albicans strains with decreased susceptibility to fluconazole, high doses of the medication should be reserved for particular situations, such as topical therapy failure or severe cases [57,71].

In pediatric patients with asplenism, vaccines against encapsulated bacteria, such as *Streptococcus pneumoniae*, *Neisseria meningitidis*, and *Haemophilus influenzae* type b, should be prescribed to prevent fulminant sepsis and death [72].

Unfortunately, the use of rhPTH as substitution treatment for pediatric patients diagnosed with primary hypoparathyroidism is not approved in Romania. Therefore, the treatment approach in this case involved administering high doses of intravenous calcium gluconate infusion to correct severe hypocalcemia and prevent the recurrence of hypocalcemic seizures. Once the serum calcium concentration improved and stabilized, the oral supplementation of calcium, along with vitamins to optimize intestinal calcium absorption, was prescribed. Additionally, oral nystatin solution was recommended to prevent systemic *Candida albicans* infections when oral candidiasis appeared.

A strict and regular follow-up plan was recommended for this patient, including periodic clinical examination and laboratory investigation assessments to detect early signs of a potential new disorder, prevent the risk of complications, and facilitate prompt medical intervention or adjustments of the prescribed treatment.

## 4. Conclusions

Hypocalcemia diagnosed in children requires a detailed evaluation of the underlying causes, especially the chronic forms, which could indicate rare conditions like genetic syndrome or autoimmune destruction responsible for its appearance.

APS-1 stands out as the most distinctive form among related syndromes diagnosed primarily in pediatric patients. It leads to chronic hypocalcemia due to autoimmune hypoparathyroidism and presents as a multisystemic disease with various affected organs and specific autoantibodies involved. Given its rarity, extensive variability in manifestations, and incomplete penetrance, APS-1 is often underdiagnosed.

The identification of *AIRE* gene mutations plays a crucial role in facilitating the genetic diagnosis, prognosis, and potential treatment of APS-1. Genetic testing enables the early detection of APS-1 and helps identify a profile of manifestations that patients may develop in the future, allowing for the prompt recognition of potentially life-threatening conditions.

Regular and stringent patient follow-up, including surveillance, medication adjustments, and the early identification of associated conditions, permits healthcare providers to establish an early diagnosis before clinical symptoms emerge, thereby preventing severe and life-threatening events.

## Figures and Tables

**Table 1:** Biological investigations performed to assess the potential occurrence of other autoimmune endocrinopathies or diseases associated with APS-1.

Biological Investigation *	Result	Reference
TSH	2.9 μIU/mL	0.66–4.14 μIU/mL
FT4	18.91 pmol/L	11.6–21.5 pmol/L
FT3	7.06 pmol/L	4.1–7.9 pmol/L
Anti-thyroid peroxidase abs	<10 UI/mL	<35.0 UI/mL
Anti-thyroglobulin abs	<1.3 UI/mL	< 4.5 UI/mL
ACTH am	29.50 pg/ml	≤46.00 pg/ml
Cortisol am	505.1 nmol/L	171–536 nmol/L
Steroid 17-hydroxylase abs	9.55	<10, GZ < 15
Steroid 21-hydroxylase abs	6.43	<10, GZ 10–15
IgA tTg abs	2.45 UI/mL	<20 UI/mL
IgG tTg abs	1.64 UI/mL	<20 UI/mL
fecal calprotectin	48.40 µg/g	<50.00 µg/g
IgG anti-Sm abs	1.4 UI/mL	<15.0 UI/mL
IgG anti-LKM1 abs	negative	negative
ANA	negative	negative
pANCA	3.5 U/mL	<7 U/mL
ASCA	1.3 U/mL	<7 U/mL
IgG ab to H(+)/K(+) ATPase	3.2 U/mL	<10.0 U/mL
IgG intrinsic factor abs	0.7 U/mL	<6.0 U/mL
folate	10.83 ng/mL	>5.38 ng/mL
ICA	0.3	<0.90
anti-GAD 2	2.3 UI/mL	<10.0 UI/mL
IA2	3.5 UI/mL	<10.0 UI/mL
B12 vitamin	763 pg/mL	211–911 pg/mL

* Abs—antibodies, TSH—thyroid stimulating hormone, FT4—free tetraiodothyronine, FT3—free triiodothyronine, ACTH—adrenocorticotropic hormone, IgA and IgG tTg abs—Ig A and Ig G anti-tissue transglutaminase antibodies, ANA—antinuclear antibody, pANCA—perinuclear antineutrophil cytoplasmic antibodies, ASCA—anti-saccharomyces cerevisiae antibodies, IgG abs to H(+)/K(+) ATPase—IgG anti-parietal cell antibodies, ICA—anti-islet cell antibodies, anti-GAD—anti-glutamic acid decarboxylase antibodies, IA2—anti-insulin autoantibodies.

## Data Availability

The data are not publicly available due to reasons of privacy.
